# Supplementary material for: The MksG nuclease is the executing part of the bacterial plasmid defense system MksBEFG
Source: Nucleic Acids Res. 2023 Mar 7;51(7):3288–306. doi: 10.1093/nar/gkad130 (PMC10123090; doi:10.1093/nar/gkad130)
Supplement: gkad130_Supplemental_File [file gkad130_supplemental_file.pdf]

Supplemental material for

The MksG nuclease is the executing part of the bacterial plasmid defense system MksBEFG

[1] Institute for General Microbiology, Christian-Albrechts-University Kiel, Am Botanischen Garten 1-9, 24118 Kiel, Germany; bramkamp@ifam.uni-kiel.de

[2] Unité de Microbiologie Structurale, Institut Pasteur, CNRS, Université Paris Cité, 75015 Paris, France; anne-marie.wehenkel@pasteur.fr

[3] Plate-forme de Cristallographie, C2RT, Institut Pasteur, CNRS UMR 3528, F-75015 Paris, France

[4] Central Microscopy Facility, Christian-Albrechts-University Kiel, Am Botanischen Garten 1-9, 24118 Kiel, Germany

Running title: The MksBEFG plasmid defense system

[#] These authors contributed equally

* To whom correspondence should be addressed: Marc Bramkamp, Christian-Albrechts-University Kiel, Institute for General Microbiology, Am Botanischen Garten 1-9, 24118 Kiel, Germany, Email: bramkamp@ifam.uni-kiel.de; Phone: +49 (0)431-880-4341; Telefax: +49(0)431-880-2198, Twitter: @BramkampLab

Table S1: Strains used in this study.

Strain Name	Description	Genotype	Reference
	Escherichia coli		
BL21 (DE3) pLysS		F– dcm ompT hsdS(rB – mB –) gal λ(DE3) [pLysS CamR]	Novagen
EMG007	BL21 (DE3) pLysS/ pET28a_His6-MksE	BL21 (DE3) pLysS derivative, pET28a mksE	This study
EMG008	BL21 (DE3) pLysS/ pET28a_His6-MksF	BL21 (DE3) pLysS derivative, pET28a mksF	This study
EMG009	BL21 (DE3) pLysS/ pET28a_His6-MksG	BL21 (DE3) pLysS derivative, pET28a mksG	This study
EMG049	BL21 (DE3) pLysS/ pET28a_*mksG*^{D279A}	BL21 (DE3) pLysS derivative, pET28a mksG^{D279A}	This study
EMG051	BL21 (DE3) pLysS *pET28a_mksG*^{E236A}	BL21 (DE3) pLysS derivative, pET28a mksG^{E236A}	This study
EMG055	*E. coli* BL21 (DE3) pLysS/ pET28a_*mksG*^{Y258A}	BL21 (DE3) pLysS derivative, pET28a mksG^{Y258A}	This study
EMG057	*E. coli* BL21 (DE3) pLysS *pET28a_mksG*^{Y276A}	BL21 (DE3) pLysS derivative, pET28a mksG^{Y276A}	This study
BL21 C41	BL21 C41 expression strain	F – ompT hsdSB (rB- mB-) gal dcm (DE3)	Sigma Aldrich
Rosetta (DE3)pLysS		F- ompT hsdSB(rB- mB-) gal dcm (DE3) pLysSRARE (CamR)	Novagen
EMG035	Rosetta (DE3) pLysS pET16b-*mksB*	Rosetta (DE3) pLysS derivative, pET16b mksB	This study
EMG041	Rosetta pLysSRARE pET16b_mksB ^{E1042Q}	Rosetta (DE3) pLysS derivative, pET16b mksB^{E1042Q}	This study
	Corynebacterium glutamicum		
MB001	MB001	ATCC 13032 with in-frame deletion of prophages CGP1 (cg1507-cg1524), CGP2 (cg1746-cg1752), and CGP3 (cg1890-cg2071)	Baumgart et al. (1)
CMG004	MB001 *ΔmksF*	MB001 derivative, *ΔmksF*	This study
CMG005	MB001 *ΔmksE*	MB001 derivative, *ΔmksE*	This study
CMG006	MB001 *ΔmksG*	MB001 derivative, *ΔmksG*	This study
CMG007	MB001 pBHK18	MB001 derivative, pBHK18	This study
CMG010	MB001 pJC1	MB001 derivative, pJC1	This study
CMG011	MB001 *ΔmksB*	MB001 derivative, *ΔmksB*	This study
CMG012	MB001 *mksG::mksG-halo-tag*	MB001 derivative, *mksG::mksG-halo-tag*	This study
CMG013	MB001 *mksE::mksE-halo-tag*	MB001 derivative, *mksE::mksE-halo-tag*	This study
CMG014	MB001 *mksF::mksF-halo-tag*	MB001 derivative, *mksF::mksF-halo-tag*	This study
CMG015	MB001 *mksB::mksB-halo-tag*	MB001 derivative, *mksB::mksB-halo-tag*	This study
CMG018	MB001 *ΔmksB mksG::mksG-halo-tag*	MB001 derivative, *ΔmksB mksG::mksG-halo-tag*	This study
CMG023	CMG011 + pBHK18	MB001 derivative, *ΔmksB* pBHK18	This study

CMG032	CMG012 + pBHK18	MB001 derivative, *mksG::mksG-halo-tag*	This study
CMG033	CMG012 + pJC1	MB001 derivative, *mksG::mksG-halo-tag*	This study
CMG034	CMG018 + pBHK18	MB001 derivative, ΔmksB *mksG::mksG-halo-tag*	This study
CMG035	CMG018 + pJC1	MB001 derivative, *ΔmksB mksG::mksG-halo-tag*	This study
CMG038	CMG011 + pJC1	MB001 derivative, *ΔmksB* pJC1	This study
CMG041	CMG004 + pBHK18	MB001 derivative, *ΔmksF* pBHK18	This study
CMG042	CMG004 + pJC1	MB001 derivative, *ΔmksF* pJC1	This study
CMG045	CMG006 + pBHK18	MB001 derivative, *ΔmksG* pBHK18	This study
CMG046	CMG006 + pJC1	MB001 derivative, *ΔmksG* pJC1	This study
RES167	RES167 *(Restriction-deficient mutant, otherwise considered wild-type)*		Tauch et al. (2)
CBK114	RES167 *mksB::mksB-mNeonGreen*	RES 167 derivative, *mksB::mksB-mNeonGreen*	Lab collection
CPF009	RES167 *mksB::mksB-mNeonGreen*, dCas-*divIVA*	RES 167 derivative, *mksB::mksB-mNeonGreen*, dCas-*divIVA,* pSG-dcas9_sgRNA-*divIVA* (IPTG-inducible)	This study

Table S2: Plasmids used in this study.

**Name**	**Description**	**Reference**
pET28a(+)	*E. coli* expression vector, carrying an N-terminal 6xHisTag/thrombin/T7Tag configuration plus an optional C-terminal HisTag sequence, Kan^r	Novagen
pMG001	pET28a(+) *mksF*	This study
pMG002	pET28a(+) *mksE*	This study
pMG003	pET28a(+) *mksG*	This study
pMG006	pET28a_*mksG*^{D279A}	This study
pMG007	*pET28a_mksG*^{E236A}	This study
pMG008	*pET28a_mksG*$^{E236A,\ D279A}$	This study
pMG017	*pET28a_mksG*^{Y258A}	This study
pMG018	*pET28a_mksG*^{Y276A}	This study
pMBA001	pET28a SUMO-*mksG*	This study
pET16b	*E. coli* expression vector, carrying an N-terminal His•Tag® sequence followed by a Factor Xa site and three cloning sites, *bla*	Novagen
pMG004	pET16b-*mksB*	This study
pMG005	pET16b_*mksB*^{E1042Q}	This study
pK19*mobsacB*	Kan^r; plasmid for allelic exchange in C. glutamicum (pK18 *oriV*$_{E.c.}$ *sacB lacZ*α)	Schäfer et al. (3)
pK19msB-Δ*mksB*	Plasmid to generate a *mksB* deletion, pK19msB-Δ*mksB*	Böhm et al. (4)
pMG009	pK19 *mobsacB ΔmksF*	This study
pMG010	pK19 *mobsacB ΔmksE*	This study
pMG011	pK19 *mobsacB ΔmksG*	This study

pMG012	pK19 *mobsacB -mksG-halo*	This study
pMG013	pK19 *mobsacB -mksE-halo*	This study
pMG014	pK19 *mobsacB -mksF-halo*	This study
pMG015	pK19 *mobsacB -mksB-halo*	This study
pJC1	*E. coli/ C. glutamicum* shuttle vector, Kan^r	Cremer et al. (5)
pBHK18	Based on pNG2 plasmid*, aph(3' (Kan^r)-IIa*	Kirchner and Tauch (6)
pXMJ19	*E. coli/ C. glutamicum* shuttle vector, p*tac*,*lacI^q*, Cam^r	Jakoby et al. (7)
pMG016	pXMJ19 *mksG*	This study
pSG-dCas9_sgRNA-*divIVA*	Ptac, lacI^q, repA, ori PUC, dCas9, *diVIVA*-sgRNA,Kan^r	Giacomelli et al. (8)

Table S3: Oligonucleotides used in this study. Specific restriction sites are underlined.

Name	Description	Sequence 5' - 3'	Restriction site
MG045	NdeI_MksF_F	TAATGCATATGACCGTTGTATCG	NdeI
MG046	MksF_BamHI_R	TACTGGATCCTCATTTATCCATCTC	BamHI
MG047	NdeI_MksE_F	GATAGCATATGAATGATCAGCTGT	NdeI
MG048	MksE_BamHI_R	CTATGGATCCTCACTTCTGTTCC	BamHI
MG049	NdeI_MksB_F	TAATGCATATGACCAGCGAACAAG	NdeI
MG050	MksB_EagI_R	ATGACGGCCGTTATTTCTCGATCC	EagI
MG051	NdeI_MksG_F	ATAGCATATGCCATTGTTTATCGAC	NdeI
MG052	MksG_BamHI_R	CGATGGATCCTCACCCACGAATT	BamHI
MG053	seq-MksB_middle1_F	AATTTTGAGTGCGAAGAGG	/
MG055	seq-MksB_middle2_F	AAGAAATCGCGCGGGAAG	/
MG057	seq-MksF_middle_F	CAGATTGAAGCGGTCCAC	/
MG058	seq-MksB_middle_R1	GTTGTTGAGCTCCAAAAGC	/
MG059	seq-MksB_middle_R2	CTCATTGGCATCGATTTG	/
MG060	SDM_MksB_G->C_E1042Q_F	CATTCTGGACcAAGCCTTCGACCGC	/
MG061	SDM_MksB_R	ACGGTGGCGTAGGTGGGA	/
MG064	HindIII_500up_mksF-ko_F	CATAAGCTTCTCGTGGGAACCCGCCAA	HindIII
MG065	500up_mksF-ko_R	GGAATGGAGTATGGAAGTTGGGCCAATTGACTATTCCAG	/
MG066	500down_mksF-ko_F	CCAACTTCCATACTCCATTCCTTTGGCAGAAAGCGAGAT	/
MG067	EcoRI_mksF_500down_R	CATGAATTCGTCTTTTTAGCTAATGAATAATCCA	EcoRI
MG080	XbaI_500up_mksE_F	CATTCTAGAGCTTCGCGATACCCGCAG	XbaI
MG081	500up_mksE-ko_R	GGAATGGAGTATGGAAGTTGGCATTCATTTATCCATCTCGC	/
MG082	mksE-ko_500down_F	CCAACTTCCATACTCCATTCCGGAAATGAAGAGGAACAGAA	/
MG083	EcoRI_mksE_500down_R	CATGAATTCGTCTTGATCAACGGGAAACAC	EcoRI
MG084	HindIII_500up_mksG_F	CATGAAGCTTACAAGCCTTCGCCCGTTATG	HindIII
MG085	500up_mksG-ko_R	GGAATGGAGTATGGAAGTTGGGTTATTTCTCGATCCTAGAGAA	/
MG086	mksG-ko_500down_F	CCAACTTCCATACTCCATTCCGTTGAAGAAATCGAGAAAGT	/
MG087	EcoRI_mksG_500down_R	CATGGAATTCGCGCCCTCCATATCGCA	EcoRI
MG088	BamHI_MksG_F	CATATGGATCCATGCCATTGTTTATC	BamHI
MG089	MksG_SalI_R	CATATGTCGACTCACCCACGAATTAC	SalI
MG090	HindIII_mksG_F	CATATAAGCTTATGCCATTGTTTATC	HindIII
MG093	pET16b_fwd	ctcgaggatccggctgcta	XhoI
MG094	pET16b_rev	catatgacgaccttcgatatgg	NdeI

MG095	mksB_fwd	atatcgaaggtcgtcatatggtgaccagcgaacaagctttag	NdeI
MG096	mksB_rev	ttagcagccggatcctcgagttatttctcgatcctagagaaactg	(XhoI)
MG097	pK19msB_fwd	GTCGACTCTAGAGGATCC	/
MG098	pK19msB_rev	CTGCAGGCATGCAAGCTT	/
MG099	mksG last 500 bp_fwd	tgattacgccaagcttgcatgcctgcagGACTTGGTGACGCCGAAG	/
MG100	mksG last 500 bp_rev	atttccatacctgcCCCACGAATTACTTTCTCGATTTC	/
MG101	halo-tag_fwd	aagtaattcgtgggGCAGGTATGGAAATCGGTAC	/
MG102	halo-tag_rev	tcgaggcggatgcgTTAGGAAATCTCCAGAGTAGAC	/
MG103	mksG 500 down_fwd	tggagatttcctaaCGCATCCGCCTCGATGTTGC	/
MG104	mksG 500 down_rev	cggtacccggggatcctctagagtcgacCACCAGCGCCGCGCCCTC	/
MG105	mksE last500bp_fwd	tgattacgccaagcttgcatgcctgcagTATCACCACAGATCAAGATGC	/
MG106	mksE last500bp_rev	atttccatacctgcCTTCTGTTCCTCTTCATTTCC	/
MG107	halo-tag_fwd	aagaggaacagaagGCAGGTATGGAAATCGGTAC	/
MG108	halo-tag_rev	gctgctttgttaaaTTAGGAAATCTCCAGAGTAGAC	/
MG109	mksE 500bp down_fwd	tggagatttcctaaTTTAACAAAGCAGCCCATG	/
MG110	mksE 500bp down_rev	cggtacccggggatcctctagagtcgacCGCATTGATGTCTTGATCAAC	/
MG111	mksF last500bp_fwd	tgattacgccaagcttgcatgcctgcagTCGCAGAGAGCCGACGCATG	/
MG112	mksF last500bp_rev	atttccatacctgcTTTATCCATCTCGCTTTCTGCCAAATC	/
MG113	halo-tag_fwd	gcgagatggataaaGCAGGTATGGAAATCGGTAC	/
MG114	halo-tag_rev	tgtttaaacccctgTTAGGAAATCTCCAGAGTAGAC	/
MG115	mksF 500bpdown_fwd	tggagatttcctaaCAGGGGTTTAAACAGTGAAG	/
MG116	mksF 500bpdown_rev	cggtacccggggatcctctagagtcgacGGTGTCTCTGTCTTTTTAGC	/
MG117	mksB last500bp_fwd	tgattacgccaagcttgcatgcctgcagACCAGCGGTGACCTGGGAAC	/
MG118	mksB last500bp_rev	atttccatacctgcTTTCTCGATCCTAGAGAAACTGGAATTG	/
MG119	halo-tag_fwd	ctaggatcgagaaaGCAGGTATGGAAATCGGTAC	/
MG120	halo-tag_rev	ctagagaaactggaTTAGGAAATCTCCAGAGTAGAC	/
MG121	mksB 500bp down_fwd	tggagatttcctaaTCCAGTTTCTCTAGGATCGAG	/
MG122	mksB 500bp down_rev	cggtacccggggatcctctagagtcgacTGGTTTTCGAGCCATTTG	/
MG127	SDM_MksG_A->C_E236A_F	CTGATGGTGGcAAACCTCGATTC	/
MG128	SDM_MksG-EA_R	AATTACTTGCGGTTCTTG	/
MG129	SDM_MksG_A->C_D279A_F	TACTGGGGTGcaCTTGACCTGG	/
MG130	SDM_MksG-DA_R	CAGCAACCGACCATTAGA	/
MG139	SDM_MksG_TAC->GCA_Y258A_F	GGGTGCGGGCgcaCGTGCAGTAG	/
MG140	SDM_MksG-Y258A_R	CAAGCAATTGTTACGCCC	/
MG141	SDM_MksG_TAC->GCA_Y276A_F	TCGGTTGCTGgcaTGGGGTGACC	/
MG142	SDM_MksG-Y276A_R	CCATTAGAAAAGTAGGGTC	/
P1		GAGGCTCACCGCGAACAGATTGGTGGCATGCCATTGTTTATCGACGA	/
P2		CTTCCTTTCGGGCTTTGTTAGCAGCCGGATCTTTACCCACGAATTACTTTCTC	/
P3		AGATCCGGCTGCTAACAAAGCCCGAAAGGAAG	/
P4		GCCACCAATCTGTTCGCGGTGAGCCTC	/

Table S4. Crystallographic data collection and refinement statistics.

**Data collection**	**MksG**
Synchrotron Beamline	SOLEIL Proxima 1
Wavelength (Å)	0.9786
Space group	$P2_12_12_1$
Cell dimensions<br>*a*, *b*, *c* (Å)	78.086, 126.776, 150.111
Resolution (Å)	61.5 – 4.6<br>(4.77 – 4.6)*
*R*pim	0.054 (0.444)
I / *s*(*I*)	9.6 (1.6)
Completeness (%)	99.5 (94.1)
CC(1/2)	0.998 (0.816)
Multiplicity	13.1 (12.7)
Total observations	114710
Unique observations	8738 (597)
**Refinement**	
Resolution (Å)	4.6
No. reflections	7594 **
*R*work/ *R*free (%)	0.27 / 0.311
No. atoms	
Protein	5862
Ligands/ions	-
Solvent	-
Average B-factors (Å^2)	
Protein	147
Ligand/ions	-
Solvent	-

R.m.s deviations	
Bond lengths (Å)	0.004
Bond angles (º)	0.888
Ramachandran favored (%)	97.4
Ramachandran outliers (%)	0.27
**PDB code**	8B7F

*Values in parenthesis correspond to the highest resolution shell.

**Number of unique reflections used for structure determination and refinement, after the merged dataset was subjected to anisotropic resolution surface cut-off with StarAniso (see Methods).

Table S5: BLI Advanced Kinetics Protocol

Step type	Sample type	Position	Duration (s)
Initial Baseline	Buffer	Tube	30
Loading of the bait	Biotinylated Protein	Drop	300
Baseline	Buffer	Tube	30
Association	Analyte (MksF)	Drop	300
Dissociation	Buffer	Tube	180

Supplementary Figures:

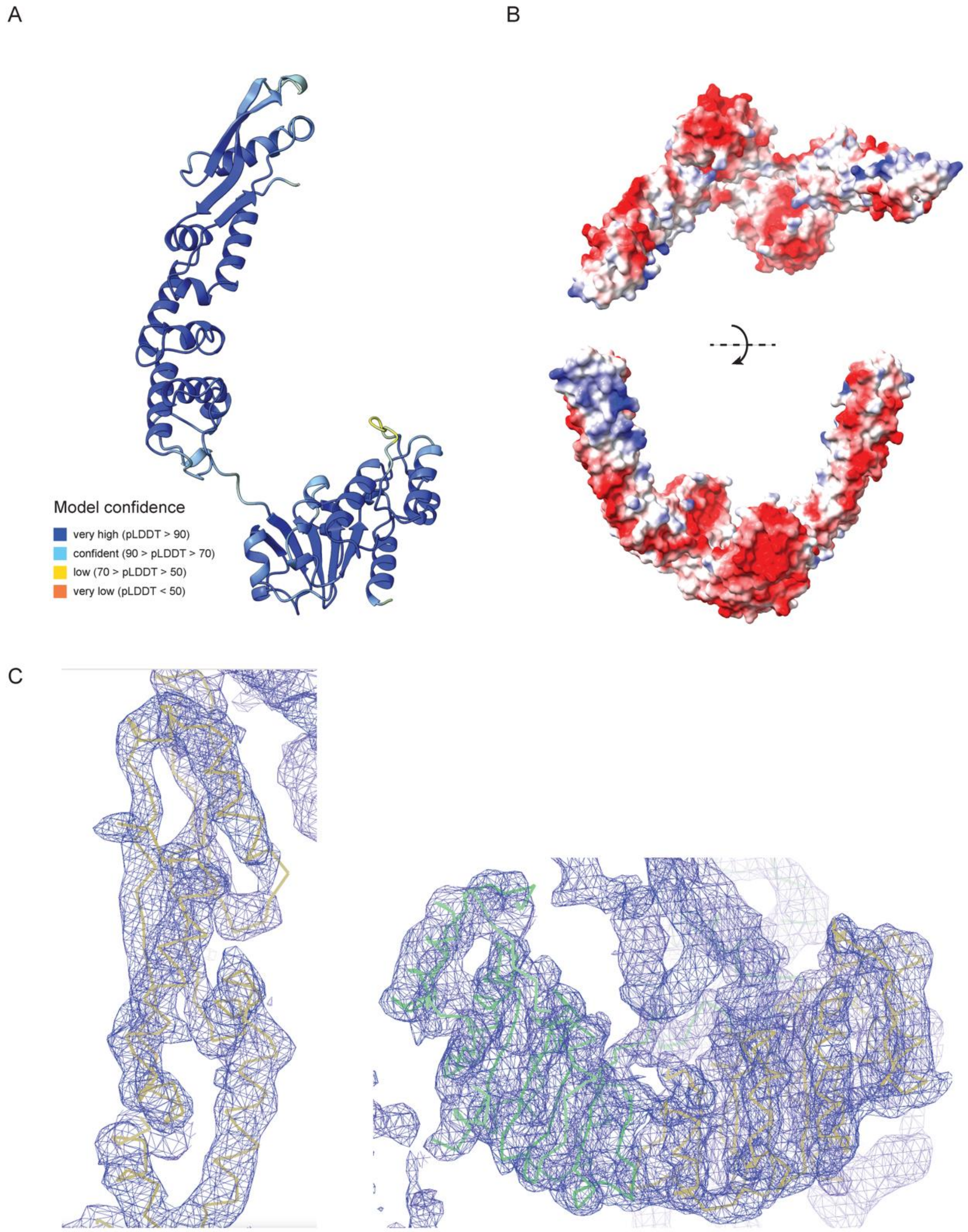

Supplementary Figure S1. Structural analysis of MksG. (A) AF model of MksG. The pLDDT confidence scores of the model are shown on the right. (B) Electrostatic surface representation generated with ChimeraX. (C) Representative regions of the final electron density map for the three crystal structures contoured at 1.35 σ, drawn with Coot.

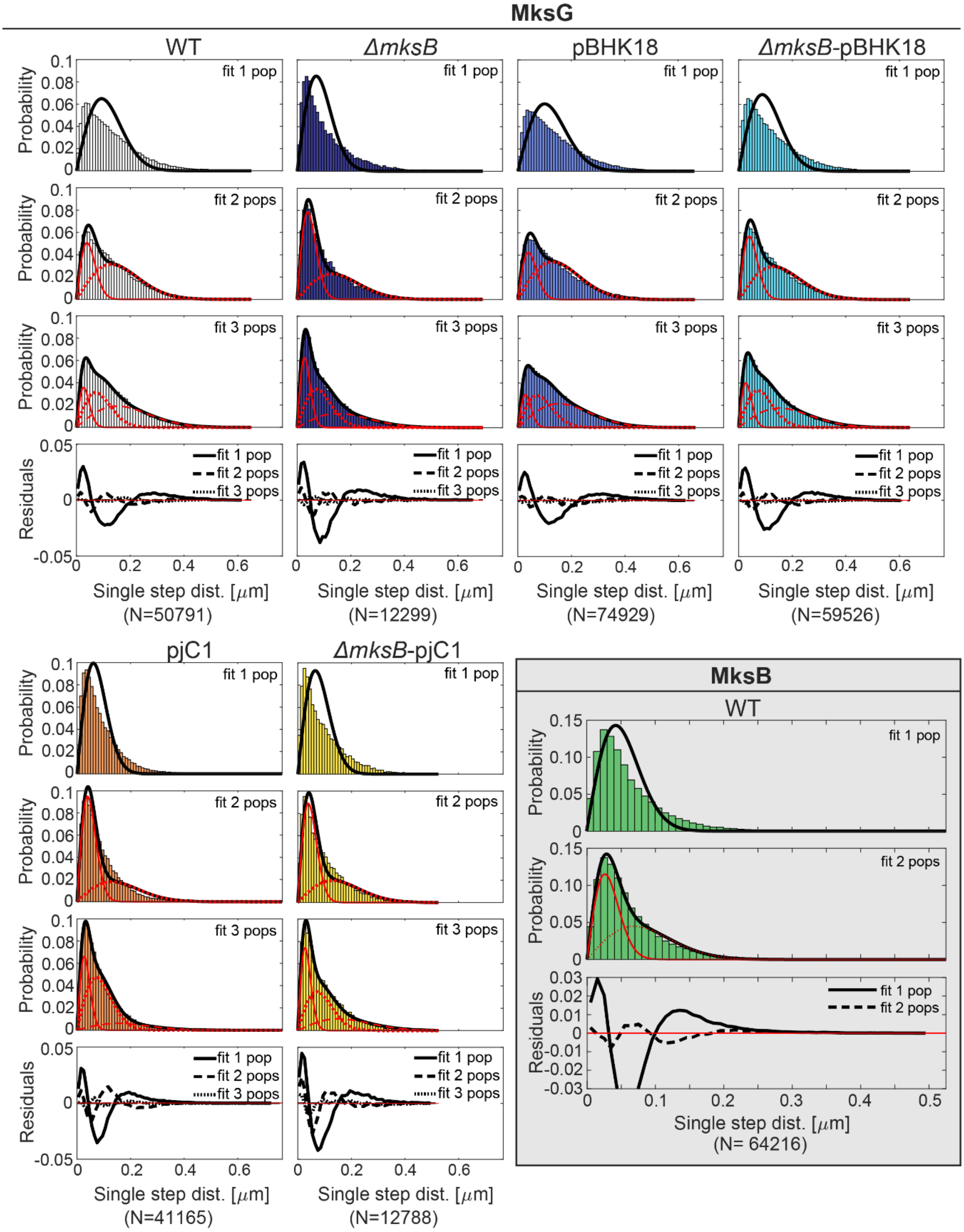

Supplementary Figure S2: Jump distance analysis of strains analyzed via SPT. The diffusion constants and associated subpopulation fractions obtained via the fitting of the cumulative distribution of the square displacements (SQD) were used to fit the jump distances of the respective strains (each subpopulation is fitted according to a rayleigh distribution). Single subpopulations are shown as red lines, while the sum of the populations is shown as a black line. Residuals obtained for the fitting of 1, 2 and 3 subpopulations are also shown to allow for visual comparison of the goodness of the fit. Jump distance analysis was performed for *C.*

glutamicum MB001 mksG-Halo (CMG012), *ΔmksB mksG-Halo* (CMG018), *mksG-Halo* + pBHK18 (CMG032), *ΔmksB mksG-Halo* + pBHK18 (CMG034), *mksG-Halo* + pJC1 (CMG033), *ΔmksB mksG-Halo* + pJC1 (CMG035) and *mksB-Halo* (CMG015).

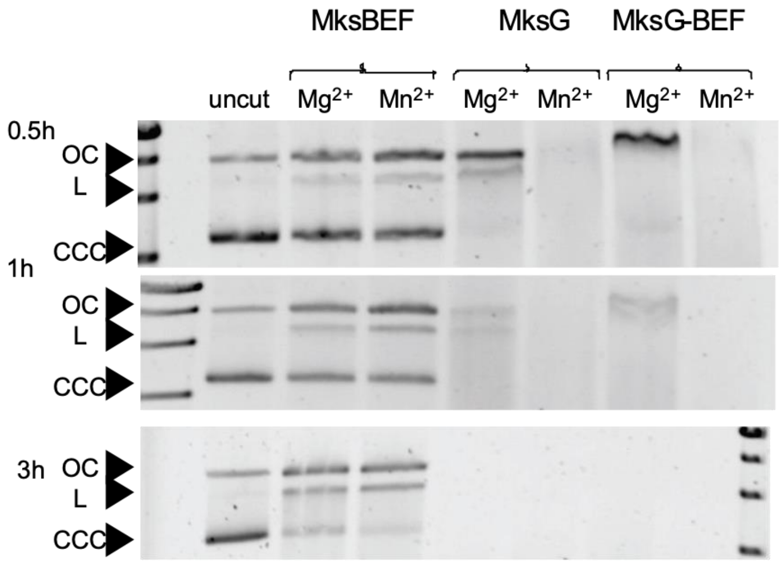

Supplementary Figure S3: Nicking Assay MksG and MksBEFG. Nuclease activity assay of MksBEF, MksG and MksBEFG, 10 µM (of each) protein were incubated for 0.5, 1, and 3h at 30°C with 250 ng plasmid DNA (pBHK18, 3,337 bp) and 10 mM Mg^{2+} or Mn^{2+}. Reactions were stopped by adding 6x purple loading dye (NEB) and boiling samples for 5 min at 90°C. DNA was separated on an agarose gel in TAE buffer and post-stained by ethidium bromide.

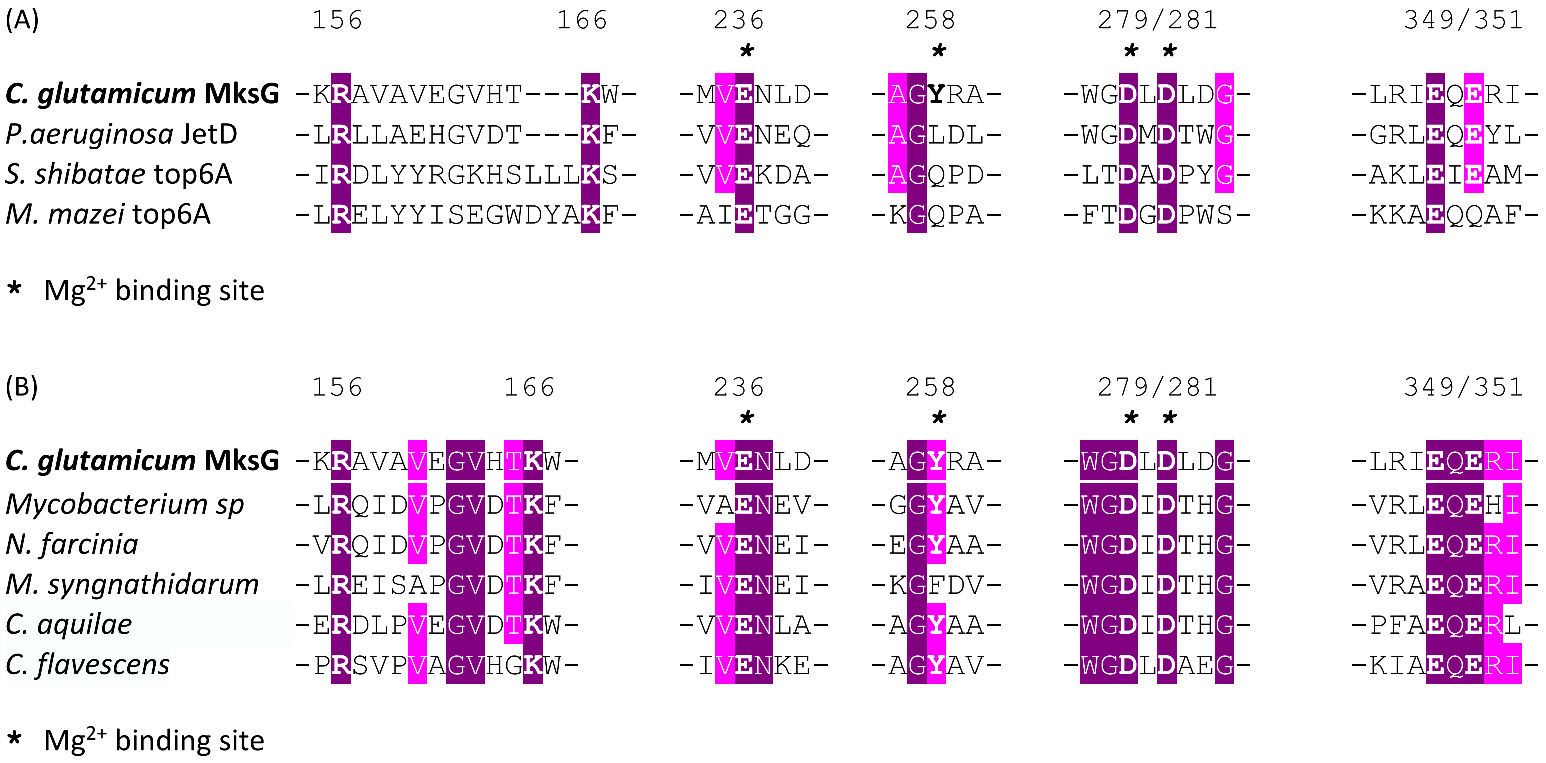

Supplementary Figure S4: Sequence alignment of MksG homologs. (A) Sequence alignment of four MksG structural homologs. Active site residues highlighted in this work are indicated with *. **(B)** Sequence alignment of Actinobacteria MksG homologs. Active site residues highlighted in this work are indicated with *. In this alignment we included the sequences extracted from a "conserved job" using the structure of JetD as working model. The chosen hits are all uncharacterized proteins from Actinobacteria species *A0A0H5P7V1_NOCFR (Nocardia farcinia); A0A1S1JWP0_9MYCO (Mycobacterium syngnathidarum); A0A1L7CDP9_9CORY (Corynebacterium aquilae); A0A1L7CLH6_CORFL (Corynebacterium flavescens)*

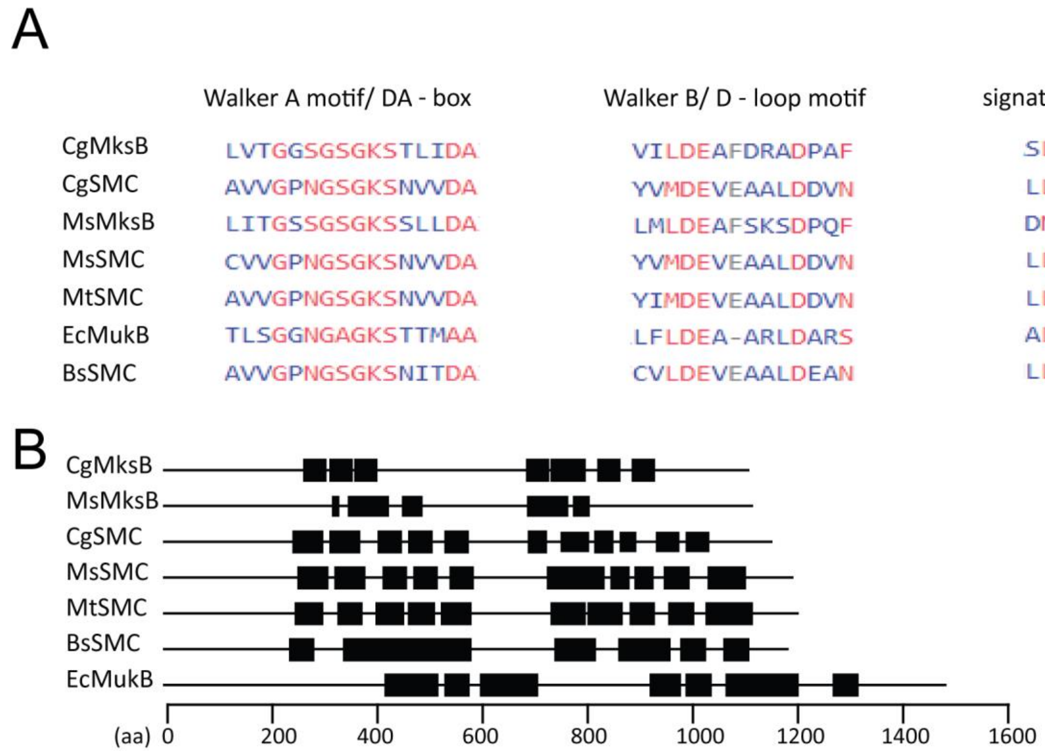

Supplementary Figure S5: Alignments of sequence motifs in condensin proteins. (A) Sequence alignment of MksB/SMC/MukB proteins form different organisms, showing the major conserved motifs. Cg, *C. glutamicum*; Ms, *M. smegmatis*; Mt, *M. tuberculosis*; Ec, *E. coli*; Bs, *B. subtlis*. Using NCBI BLASTp (10) (B) Coilel-coild prediction of MksB/SMC/MukB proteins using Coils program (https://mybiosoftware.com/coils-2-2-prediction-coiled-coil-regions-proteins.html) (11). Black squares represent coils. The bottom axis shows the position of the amino acid (aa) corresponding to the coiled-coil segment within a protein's primary structure.

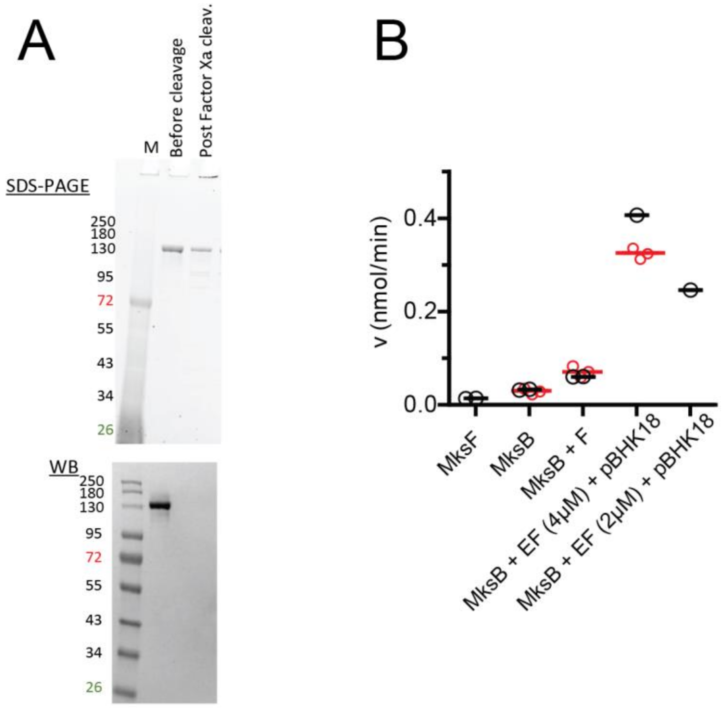

Supplementary Figure S6: Cleavage of His Tag from MksB. (A) His Tag of MksB was cleaved using Factor Xa protease. The process of HisTag cleavage was analyzed using SDS-PAGE (stain-free) and Western blotting (WB) with anti-His antibody. MksB monomer with HisTag has a molecular weight of 126.61 kDa and without the tag 124.22 kDa. (B) ATPase activity analysis of MksB without the His Tag. $MksB_2$ (2 µM) and 4 µM of other subunits were assayed (unless stated otherwise). Plasmid DNA concentration was 50 ng $µl^{-1}$ and 2 mM Mg-ATP were used. ATPase measurements were taken every minute in a time-course of 3 h, no substrate controls and ATP auto-hydrolysis were subtracted from values. Each data point represents one time-course measurement, the mean is shown as line with standard error, n=2; MksB+ EF + pBHK18, n=1). In red, we show the exact same measurements from main Figure 4C as an overlay.

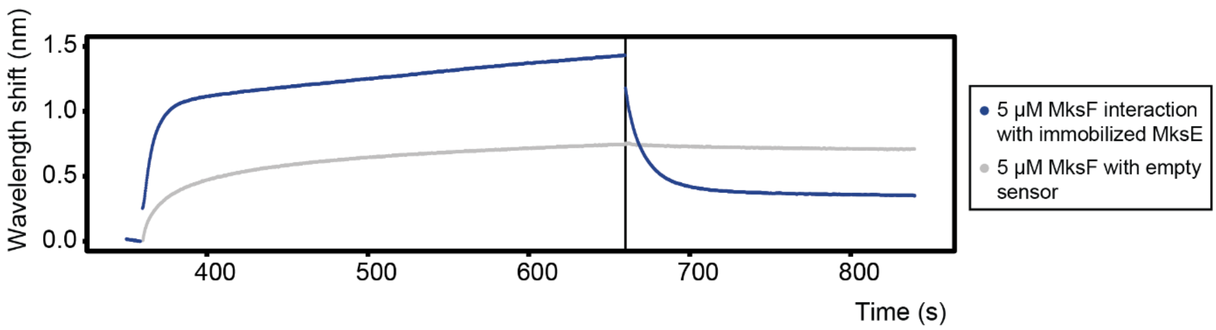

Supplementary Figure S7: Bio Layer Interferometry test for unspecific binding of MksF. The blue curve shows the protein-protein interaction between the immobilized MksE with MksF (5 µM) (main Figure 5A). The grey curve shows minor unspecific binding of unbiotinylated MksF protein to an empty SA-biosensor.

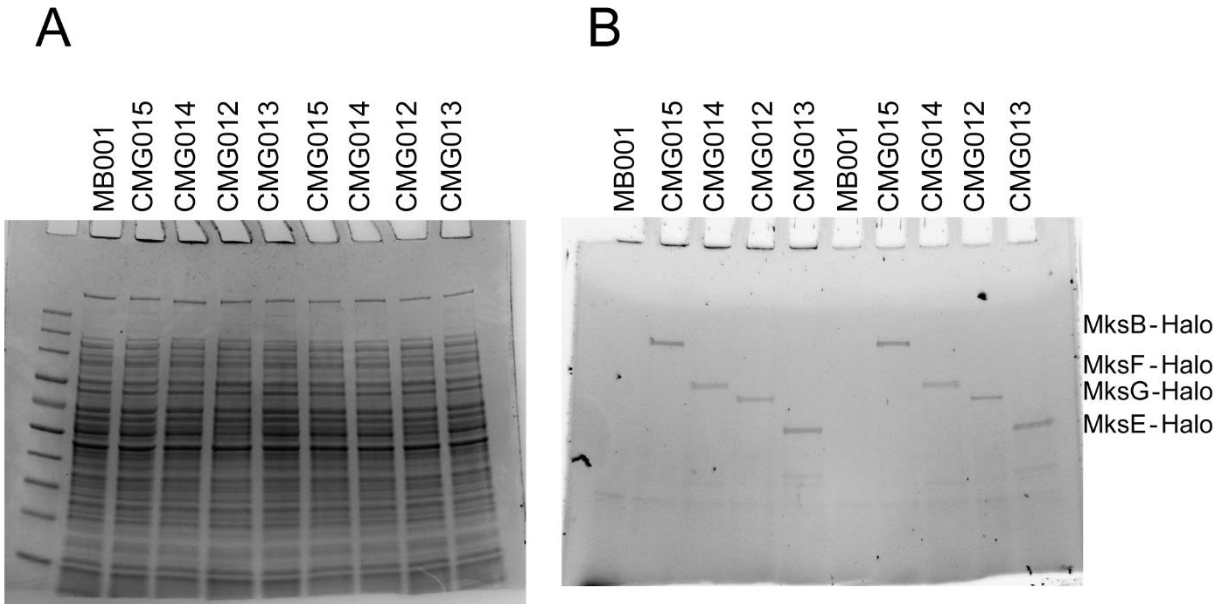

Supplementary Figure S8: SDS-PAGE analysis of allelic replacement strains with Halo-Tag. Strains were grown for 5 h in BHI medium, OD_{600} was adjusted to OD=15/ml, washed once in PBS buffer. Cells were stained 15 mins with 5 µM TMR dye, washed again once with PBS buffer. Cells were disrupted by sonication. Samples were mixed with 4x SDS-loading dye and heated for 10 min at 40 °C. 20 µl/ lane were applied. (A) Coomassie-stained gel; lane 1, marker (NEB, Color pre-stained protein standard), lane 2-6, lysed cells; lane 7-10, cleared lysates. (B) In-gel fluorescence; lane 1-5, lysed cells; lane 6-10, cleared lysates.

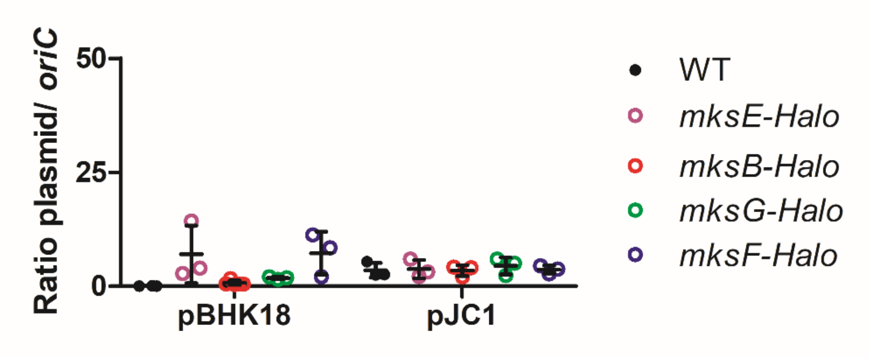

Supplementary Figure S9: qPCR analysis of strains with Halo-fusion. Plasmid copy numbers of low copy (pBHK18) and high copy number plasmids (pJC1) relative to *oriC* numbers per cell, assayed by qPCR. Ratios were compared between *C. glutamicum* WT (MB001), *mksE-Halo*, *mksB-Halo*, *mksG-Halo* and *mksF-Halo* cells grown in BHI medium with selection antibiotic (mean ± SD, n =3).

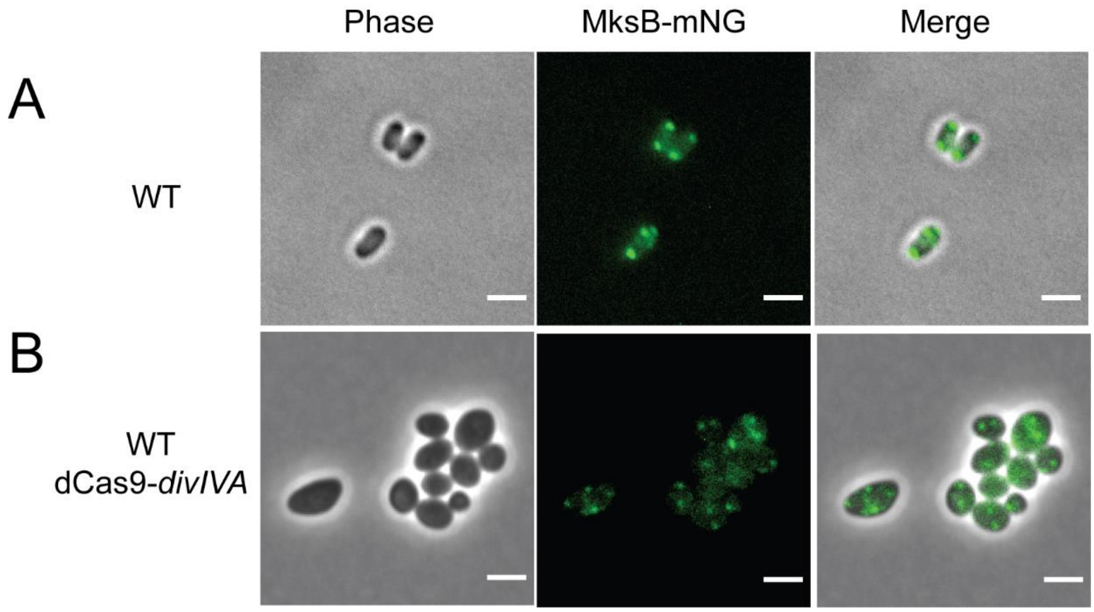

Supplementary Figure S10: DivIVA-depletion leads to mislocalization of MksB. Microscopic images of strain (CPF009) expressing MksB-mNeonGreen (mNG) uninduced (A) and CRISPRi/dCas9 for DivIVA-depletion upon induction (B). MksB-mNG foci are shown in green. Scale bar, 2 µm.

Supplementary References:

1. Baumgart, M., Unthan, S., Ruckert, C., Sivalingam, J., Grunberger, A., Kalinowski, J., Bott, M., Noack, S. and Frunzke, J. (2013) Construction of a prophage-free variant of *Corynebacterium glutamicum* ATCC 13032 for use as a platform strain for basic research and industrial biotechnology. *Appl Environ Microbiol*, **79**, 6006-6015.
2. Tauch, A., Kirchner, O., Loffler, B., Gotker, S., Puhler, A. and Kalinowski, J. (2002) Efficient electrotransformation of *Corynebacterium diphtheriae* with a mini-replicon derived from the *Corynebacterium glutamicum* plasmid pGA1. *Curr Microbiol*, **45**, 362-367.
3. Schäfer, A., Tauch, A., Jäger, W., Kalinowski, J., Thierbach, G. and Pühler, A.J.G. (1994) Small mobilizable multi-purpose cloning vectors derived from the *Escherichia coli* plasmids pK18 and pK19: selection of defined deletions in the chromosome of *Corynebacterium glutamicum*. **145**, 69-73.
4. Böhm, K., Giacomelli, G., Schmidt, A., Imhof, A., Koszul, R., Marbouty, M. and Bramkamp, M. (2020) Chromosome organization by a conserved condensin-ParB system in the actinobacterium *Corynebacterium glutamicum*. *Nat Commun*, **11**, 1485.
5. Cremer, J., Eggeling, L., Sahm, H.J.M. and MGG, G.G. (1990) Cloning the *dapA dapB* cluster of the lysine-secreting bacterium *Corynebacterium glutamicum*. **220**, 478-480.
6. Kirchner, O. and Tauch, A. (2003) Tools for genetic engineering in the amino acid-producing bacterium *Corynebacterium glutamicum*. *Journal of Biotechnology*, **104**, 287-299.
7. Jakoby, M., Ngouoto-Nkili, C.-E. and Burkovski, A.J.B.T. (1999) Construction and application of new *Corynebacterium glutamicum* vectors. **13**, 437-441.

8. Giacomelli, G., Feddersen, H., Peng, F., Martins, G.B., Grafemeyer, M., Meyer, F., Mayer, B., Graumann, P.L. and Bramkamp, M. (2022) Subcellular Dynamics of a Conserved Bacterial Polar Scaffold Protein. *Genes (Basel)*, **13**.
9. Lu, S., Wang, J., Chitsaz, F., Derbyshire, M.K., Geer, R.C., Gonzales, N.R., Gwadz, M., Hurwitz, D.I., Marchler, G.H., Song, J.S. *et al.* (2020) CDD/SPARCLE: the conserved domain database in 2020. *Nucleic Acids Res*, **48**, D265-D268.
10. Papadopoulos, J.S. and Agarwala, R. (2007) COBALT: constraint-based alignment tool for multiple protein sequences. *Bioinformatics*, **23**, 1073-1079.
11. Lupas, A., Van Dyke, M. and Stock, J. (1991) Predicting Coiled Coils from Protein Sequences. **252**, 1162-1164.
